# The role of intelligence in decision‐making in early adolescence

**DOI:** 10.1111/bjdp.12261

**Published:** 2018-08-19

**Authors:** Eirini Flouri, Vanessa Moulton, George B. Ploubidis

**Affiliations:** ^1^ Department of Psychology and Human Development UCL Institute of Education University College London UK; ^2^ Centre for Longitudinal Studies UCL Institute of Education University College London UK

**Keywords:** Cambridge Gambling Task, decision‐making, intelligence, IQ, Millennium Cohort Study

## Abstract

This study investigated the role of intelligence and its development across childhood in decision‐making in adolescence (age 11 years). The sample was 12,514 children from the UK's Millennium Cohort Study, followed at ages 3, 5, 7, and 11 years. Decision‐making (risk‐taking, quality of decision‐making, risk adjustment, deliberation time, and delay aversion) was measured with the Cambridge Gambling Task. Even after adjustment for confounding, intelligence was positively associated with risk adjustment and quality of decision‐making in both boys and girls. Furthermore, in girls risk adjustment was related positively to IQ gains. Our findings suggest that there are important, substantively, associations between intelligence and adapting behaviour to risk at the cusp of adolescence, the period when the response to risk can shape life trajectories.

**Statement of Contribution**

***What is already known on this subject***

- In children, intelligence and decision‐making, measured with gambling tasks, are inconsistently linked.
- This could be due to gambling tasks not separating risk‐taking from contingency‐learning.

***What the present study adds***

- This study measured 11‐year‐olds’ decision‐making using a gambling task in which probabilities of different outcomes are presented explicitly.
- IQ was positively associated with risk adjustment and quality of decision‐making.
- Also significant were IQ gains (for risk adjustment, only in girls).
- There are links between intelligence and adapting behaviour to statistical risk in children.

## Background

At least intuitively, decision‐making and intelligence should be inter‐related. However, empirical studies in cognitive psychology, which typically infer decision‐making by performance in gambling tasks, have shown that this relationship is complex in both adults (Toplak, Sorge, Benoit, West, & Stanovich, 2010; Webb, DelDonno, & Killgore, 2014) and children (Crone & van der Molen, 2004; Lehto & Elorinne, 2003; Li *et al*., 2017; Smith, Xiao, & Bechara, 2012). With adults, studies tend to show relationships in clinical populations – perhaps reflecting the presence of multiple deficits in their functioning (Toplak *et al*., 2010) – and only an asymmetric dependence in the general population, such that general cognitive ability is not dependent on the intactness of decision‐making, but decision‐making is influenced by the intactness of cognitive ability. That is, while one can have normal cognitive ability in the presence or absence of deficits in decision‐making, decision‐making is worse in the presence of low cognitive ability (Bechara, Damasio, Tranel, & Anderson, 1998). With children and adolescents, studies showing independence of intelligence and decision‐making typically attribute it to the faster pace of maturation of the ventromedial prefrontal cortex compared to that of the dorsolateral prefrontal cortex (Steinberg, 2005 for a review). The ventromedial prefrontal cortex is activated during gambling tasks, that is when individuals make choices that they are uncertain about (e.g. when guessing) and that involve rewards and punishments based on those choices. The dorsolateral prefrontal cortex is activated in executive function tasks, strongly linked to intelligence (Brydges, Reid, Fox, & Anderson, 2012).

However, there may be another reason for this complexity. In most empirical studies to date, decision‐making was measured with the Iowa Gambling Task (IGT) (Bechara, Damasio, Damasio, & Anderson, 1994) in which participants are unaware of the probabilities of the contingencies when they start performing at the beginning of the task. With this task, therefore it is difficult to distinguish risk‐taking from contingency learning. Despite this, some research using the IGT has shown that successful performance is driven by cognitive rather than by more implicit, emotion‐based processes in both adults (Webb *et al*., 2014) and children (Li *et al*., 2017). That is, although performance on the IGT is typically interpreted as reflecting ‘hot’ (affective) decision‐making, it appears that it is also linked with deliberate, cognitive capacities (associated with intelligence). Building on this observation, we carried out this study to investigate whether intelligence in childhood predicts adolescent decision‐making measured with a gambling task (the Cambridge Gambling Task (CGT); Rogers *et al*., 1999) in which probabilities of different outcomes are presented explicitly. We also explored the role of the childhood trajectory of intelligence in adolescent decision‐making to assess whether the level of or changes in intelligence are related to decision‐making. We expected that intelligence would be associated with the aspects of ‘rational’ decision‐making on the CGT (such as risk adjustment and quality of decision‐making, see ‘Measures’) where participants must effectively make judgements about rewards and losses, as well as factor uncertainty of outcomes and risk. These are therefore situations where responses are expected to be driven by ‘cold’, data‐oriented cost/benefit analysis, associated with intelligence.

To test our expectation, we used data from the Millennium Cohort Study (MCS), a birth cohort of over 19,000 children in the United Kingdom (www.cls.ioe.ac.uk/mcs). Our analysis was carried out in two steps. First, we described the trajectory (level and rate of change over time) of intelligence in childhood (from age 3 years, when intelligence was first measured in MCS, until age 11 years). Second, we examined whether it was related to decision‐making at age 11 even after controlling for variables likely to confound the association between intelligence and decision‐making in children, such as parental education, ethnicity, exact age, and emotional and behavioural problems (Bubier & Drabick, 2008; Flouri, Ruddy, & Midouhas, 2017). All analyses were stratified by gender in view of the evidence for gender differences in both intelligence (Arden & Plomin, 2006) and decision‐making (Steinberg, 2008) in children.

## Method

### Sample

The Millennium Cohort Study (MCS) is a population‐based cohort of children born in the United Kingdom over 12 months from 1 September 2000. The sample selected was clustered geographically, and was disproportionately stratified to over‐represent areas with high proportions of ethnic minorities in England, residents of areas of high child poverty and residents of the three smaller countries of the United Kingdom. In MCS, intelligence in childhood was measured at ages 3, 5, 7, and 11. The analytic sample was children (singletons and first‐born twins or triplets) with intelligence data in at least one of these ages and with at least one measure of decision‐making at age 11 (*N* = 12,514, 50.18% male). Ethical approval for MCS, a generally available data set, was gained from NHS Multi‐Centre Ethics Committees, and parents gave informed consent before interviews took place.

### Measures

*Intelligence (IQ)* was calculated for ages 3, 5, 7, and 11 using multiple age‐adjusted ability scores. At age 3, ability was assessed with the Bracken School Readiness Assessment‐Revised, which measures children's ‘readiness’ for formal education by testing their knowledge and understanding of basic concepts (Bracken, 1998), and the second edition of the British Ability Scales (BAS; Elliott, Smith, & McCulloch, 1996) for Naming Vocabulary, which measures expressive language. At age 5, ability was assessed with BAS Naming Vocabulary, BAS Pattern Construction (measuring spatial problem‐solving), and BAS Picture Similarities (measuring non‐verbal reasoning). At age 7, it was measured with BAS Pattern Construction, BAS Word Reading (measuring educational knowledge of reading), and the National Foundation for Educational Research Progress in Maths. At age 11, it was measured with BAS Verbal Similarities, which assesses verbal reasoning and verbal knowledge.

When multiple cognitive assessments were available (i.e. at ages 3, 5, and 7), IQ at each age was measured using the scores derived from a factor analysis of the available assessments at that age. Within each age, the first factor loading was the verbal assessment (i.e. BAS Naming Vocabulary at ages 3 and 5 and BAS Word Reading at age 7), which was ‘linearly stretched’ as appropriate to ensure that measurement scales were comparable across ages (de Jonge, Veenhoven, & Arends, 2014).^1^ Then, the score of the factor within each age was transformed into a standardized score with a mean of 100 and a standard deviation of 15 (Hanscombe *et al*., 2012). Multiple well‐validated assessments are thought to measure the underlying general intelligence factor (‘g’), which, at least in adults, has been shown not to be dependent on the use of specific mental ability tasks (Johnson, Bouchard, Krueger, McGue, & Gottesman, 2004). Therefore, the comparability of IQ over time can only be theoretically inferred here, in line with previous research on the trajectories of ‘g’ in children (von Stumm & Plomin, 2015).

Decision‐making was measured with the Cambridge Gambling Task at age 11. This task is a measure of decision‐making abilities with the advantage of assessing different aspects of decision‐making separately, for example risky/rational choices, betting behaviour, reaction time, risk adjustment (Deakin, Aitken, Robbins, & Sahakian, 2004; Rogers *et al*., 1999), and all that outside a learning context (Rogers *et al*., 1999). Participants face all relevant information explicitly, allowing for the different components of decision‐making to be measured in standardized conditions (Deakin *et al*., 2004; Rogers *et al*., 1999). The task measures several aspects of decision‐making, including risk‐taking through betting behaviour (participants are presented with explicit probabilities and asked to choose the more likely option and then to determine the magnitude of bet they are willing to risk given the probabilities) as follows: participants are presented with a row of ten boxes (red and blue) across the top of a computer screen and are told that the computer has hidden a ‘token’ behind one of them. They have to choose a) what colour of box they believe the token is hidden behind (red or blue) and b) the number of accumulated points they want to gamble on having made the correct colour choice. The proportion of red to blue boxes (box ratio) is varied over the task pseudo‐randomly to assess the influence of statistical risk on decision‐making. Participants start with 100 points and select a proportion of these points to bet on their decision. A circle in the centre of the screen displays the current bet value, which either increases or decreases incrementally (depending on the task variant selected). Participants press this button when it shows the proportion of their score they would like to bet. These points are either added to, or taken away from, their total score, depending on their decision and where the token was actually hidden.

The task produces six outcome measures. *Risk‐taking* (usually taken to approximate thrill‐seeking) is the mean proportion of points bet on trials where the most likely outcome was chosen. *Quality of decision‐making* is the mean proportion of trials where the correct colour outcome was selected. *Deliberation time* is the mean time taken to make a box colour response. *Risk adjustment* is the extent to which betting behaviour is moderated by the box ratio and reflects the tendency to stake higher bets on favourable compared to unfavourable trials. *Delay aversion* (seen as a driver of impulsivity) is the difference in percentage bet in ascending versus descending conditions. Finally, o*verall proportion bet* is the mean proportion of points bet across all trials.

The following variables were considered as potential confounders (associated with both intelligence and decision‐making in children): ethnicity (White, Black, Indian, Pakistani/Bangladeshi, mixed, and other), exact age, maternal education (National Vocational Qualification levels 1–5, overseas qualification, or no qualification) by the end of the study period (age 11), and internalizing and externalizing problems at age 11, measured with the main respondent's report of the Strengths and Difficulties Questionnaire (Goodman, 1997). Externalizing problems (α  =  0.81) comprised conduct problems and hyperactivity, and internalizing problems (α = 0.76) comprised emotional symptoms and peer problems.

### Analysis plan

**Figure 1:**
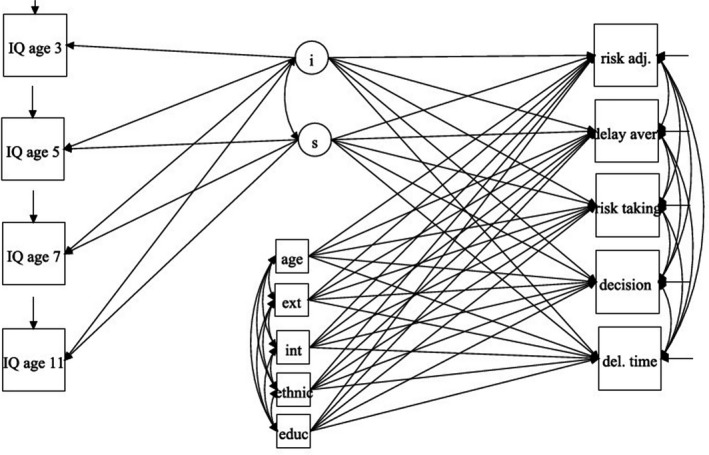
The model fitted to predict decision‐making from IQ. *Note*. i = intercept, s = slope, age = exact age, ext = externalizing problems, int = internalizing problems, ethnic = ethnicity, educ = mother's education, risk adjust = risk adjustment, delay aver = delay aversion, risk taking = risk‐taking, decision = quality of decision‐making, del. time = deliberation time. Overall proportion bet is excluded from the model due to its very strong correlation with risk‐taking (see main text for details).

To describe how intelligence develops across childhood, we fitted a latent growth curve (LGC) model. We allowed the growth parameters (intercept and slope) to correlate with each other. The intercept is the value (score) at the starting point, and the slope is the rate of change in the score with time. The slope therefore represents linear (constant) changes. At each age, loadings on the slope were set to 0, 1, 2, 4, representing 2‐year periods from the baseline. Therefore, the intercept was set where the slope had a zero loading. To explore how this development predicts decision‐making, we then specified the intercept and slope of intelligence to predict, in a regression model, the decision‐making variables, which were allowed to correlate with each other (Figure 1). We estimated a multigroup model to test for effects separately in boys and girls, and adjusted for confounders. Multiple imputation was used to handling missing data on the explanatory variables (i.e. the covariates). Twenty‐five imputed data sets were generated using sequential regression models (Asparouhov & Muthen, 2010). Full information maximum likelihood was used to deal with missing data on the dependent variables. All models were fitted in Mplus 7.0 (Muthén & Muthén, 1998). In view of the large sample size and the number of comparisons made, significance was set at *a* = .01.

## Results

The LGC model fitted the data well, χ² (10) = 291.63, RMSEA = .067 CFI = .937, TLI = .925. We found evidence for the type of gender differences in cognitive trajectories documented in previous research with children (von Stumm & Plomin, 2015), with girls starting with higher IQ but boys steadily catching up. In our sample, girls’ scores also showed almost double the variance in the slope. In addition, compared to boys’ scores which appeared to improve with age, girls’ declined, and at twice the rate. For both genders, the intercept–slope covariance was negative, suggesting that those with higher intercept estimates tended to have smaller slope estimates. (Boys: *N* = 6279, i = 100.21 (.36), variance = 124.41, s = 0.20 (.06), variance = 0.35; girls: *N* = 6235, i = 103.59 (.39), variance = 107.34, s = −0.45 (.06), variance = 0.64.)

Next, we examined the correlations between all the study variables (Table 1). As expected, our confounders were, in general, related to both intelligence and decision‐making. Intelligence, related modestly to decision‐making in general, was most strongly associated with risk adjustment, indicating that children with a higher IQ tended to adjust their risk‐taking more in response to the changing likelihood of a win, and quality of decision‐making. Children, especially boys, with a higher IQ were also quicker at making decisions. Intelligence was also inversely correlated with risk‐taking in both genders and with delay aversion in girls.

**Table 1:** Pairwise Pearson's correlations of study variables and descriptive statistics (unweighted data)

	1	2	3	4	5	6	7	8	9	10	11a	11b	11c	11d	11e	11f	12a	12b	12c	12d	12e	12f	12g	13	14
1. IQ, age 3		.46^a^	.40^a^	.32^a^	−.07^a^	.13^a^	−.06^a^	.14^a^	−.03	−.04^a^	.28^a^	−.01	−.08^a^	−.28^a^	−.11^a^	−.06^a^	.10^a^	.18^a^	.04^a^	−.02	−.09^a^	−.10^a^	−.21^a^	−.19^a^	−.15^a^
2. IQ, age 5	.47^a^		.60^a^	.41^a^	−.09^a^	.15^a^	−.08^a^	.17^a^	−.03	−.08^a^	.24^a^	.02	−.05^a^	−.25^a^	−.10^a^	−.05^a^	.11^a^	.19^a^	.02	−.02	−.09^a^	−.07^a^	−.23^a^	−.22*	−.17^a^
3. IQ, age 7	.40^a^	.58^a^		.41^a^	−.08^a^	.21^a^	−.14^a^	.22^a^	−.07^a^	−.07^a^	.10^a^	.01	.01	−.13^a^	−.05^a^	.00	.11^a^	.20^a^	.04^a^	−.04^a^	−12^a^	−.05^a^	−.23^a^	−.29^a^	−.21^a^
4. IQ, age 11	.32^a^	.39^a^	.40^a^		−.07^a^	.10^a^	−.00	.14^a^	−.03	−.10^a^	.10^a^	.02	.03^a^	−.17^a^	−.00	−.02	.11^a^	.18^a^	.02	−.03^a^	−.08^a^	−.04^a^	−.20^a^	−.25^a^	−.17^a^
5. Risk‐taking	−.10^a^	−.11^a^	−.08^a^	−.09^a^		.10^a^	−.04	−.20^a^	.02	−.01	−.08^a^	.03	.03^a^	.06^a^	.03	.00	−.02	−.04^a^	−.01	.01	.01	.01	.03^a^	.10^a^	.01
6. Quality of decision‐making	.09^a^	.15^a^	.19^a^	.13^a^	.13^a^		−.21^a^	.30^a^	−.07^a^	.03	.03	−.01	.03	−.02	−.04^a^	.00	.05^a^	.09^a^	.01	−.04^a^	−.03	.01	−.10^a^	−.12^a^	−.10^a^
7. Deliberation time (milliseconds)	−.02	−.06^a^	−.09^a^	.02	−.05^a^	−.18^a^		−.06^a^	−.16^a^	.03	.03	−.01	−.03	−.01	−.01	−.01	−.02	−.04^a^	−.01	−.00	.04^a^	−.01	.04^a^	.06^a^	.06^a^
8. Risk adjustment	.12^a^	.16^a^	.19^a^	.16^a^	−.18^a^	.25^a^	−.02		−.17^a^	.02	.06^a^	.01	.01	−.05^a^	−.06^a^	−.01	.05^a^	.10^a^	.02	−.02	−.06^a^	−.03	−.08^a^	−.14^a^	−.09^a^
9. Delay aversion	−.05^a^	−.06^a^	−.05^a^	−.07^a^	.27^a^	−.06^a^	−.13^a^	−.16^a^		−.02	.01	−.02	.01	−.01	.01	−.02	−.00	−.02	.01	.02	.03	−.03	−.01	.06^a^	.04^a^
10. Exact age	−.02	−.07^a^	−.06^a^	−.09^a^	.00	.01	.00	.03^a^	−.02		.01	−.00	−.00	.02	−.03^a^	−.01	.00	−.02	.00	.01	−.01	−.00	.02	−.04^a^	.01
11. Ethnicity																									
a. White	.32^a^	.22^a^	.10^a^	.08^a^	−.12^a^	.00	.03	.07^a^	−.02	.03							−.02	.08^a^	.03	.10^a^	.04^a^	−.19^a^	−.17^a^	−.01	−.04^a^
b. Mixed	−.02	.02	−.00	.01	.02	.03	.01	−.00	−.01	.01							.05^a^	−.01	−.01	−.02	−.01	.01	.01	.02	.01
c. Indian	−.07^a^	−.04^a^	.02	.04^a^	.03	.02	.02	.01	.01	−.00							.03	−.02	.01	−.04^a^	−.01	.05^a^	.03	−.01	.01
d. Pakistani/Bangladeshi	−.31^a^	−.23^a^	−.13^a^	−.15^a^	.09^a^	−.01	−.05^a^	−.08^a^	.02	.03^a^							−.04^a^	.10^a^	−.02	−.05^a^	−.01	.17^a^	.18^a^	.02	.05^a^
e. Black	−.12^a^	−.09^a^	−.06^a^	.01	.06^a^	−.04^a^	.00	−.04^a^	.01	−.01							.03	.01	−.01	−.07^a^	−.03	.08^a^	.05^a^	−.02	−.02
f. Other	−.09^a^	−.05^a^	.01	.01	.03	.01	−.02	−.00	.00	−.02							.01	.00	−.02	−.03	−.02	.05^a^	.05^a^	−.02	.03
12. Mother's education																									
a. NVQ5	.11^a^	.12^a^	.14^a^	.12^a^	−.05^a^	.06^a^	−.01	.07^a^	−.00	.01	.01	.03	.04^a^	−.05^a^	.01	−.01								−.09^a^	−.06^a^
b. NVQ4	.18^a^	.16^a^	.17^a^	.17^a^	−.03^a^	.06^a^	−.02	.06^a^	−.05^a^	−.00	.06^a^	−.00	−.01	−.11^a^	.04^a^	−.01								−.14^a^	−.10^a^
c. NVQ3	.03	.04^a^	.05^a^	.04^a^	−.00	−.00	−.01	.01	−.04^a^	−.00	.02	−.00	.01	−.02	−.03^a^	.03								−.02	−.02
d. NVQ2	−.01	−.01	−.06^a^	−.05^a^	−.01	−.01	.01	−.02	.02	−.01	.11^a^	−.01	−.04^a^	−.07^a^	−.06^a^	−.04^a^									.00
e. NVQ1	−.07^a^	−.08^a^	−.11^a^	−.08^a^	.01	−.04^a^	.01	−.02	.03	.00	.05^a^	.00	−.03	−.02	−.03^a^	−.04^a^								.04^a^	.04^a^
f. Overseas qualification	−.13^a^	−.06^a^	−.01	−.01	.02	.01	−.01	−.00	−.01	−.01	−.19^a^	.01	.08^a^	.15^a^	.05^a^	.09^a^								.08^a^	.03^a^
g. No qualification	−.24^a^	−.24^a^	−.22^a^	−.20^a^	.07^a^	−.08^a^	.00	−.09^a^	.04^a^	.01	−.19^a^	.01	.01	.22^a^	.05^a^	.03							−	.02	.14^a^
13. Externalizing, age 11	−.21^a^	−.21^a^	−.29^a^	−.23^a^	.10^a^	−.12^a^	.06^a^	−.13^a^	.12^a^	−.03	.01	.01	−.02	.02	−.03^a^	−.01	−.09^a^	−.15^a^	−.05^a^	.06^a^	.11^a^	−.00	.14^a^	.15^a^	.50^a^
14. Internalizing, age 11	−.17^a^	−.17^a^	−.23^a^	−.19^a^	.04^a^	−.07^a^	.06^a^	−.08^a^	.04^a^	−.02	−.04^a^	.02	−.02	.07^a^	−.01	.00	−.07^a^	−.11^a^	−.03^a^	.04^a^	.07^a^	.03	.10^a^	.49^a^	
Boys																									
*N*	5,274	5,759	5,432	6,225	6,278	6,279	6,279	6,277	6,239	6,279	6,279	6,279	6,279	6,279	6,279	6,279	6,260	6,260	6,260	6,260	6,260	6,260	6,260	6,033	6,043
Mean	98.85	99.86	100.5	100.6	.576	.799	3262	.662	.314	10.67	.833	.026	.026	.068	.033	.014	.078	.280	.086	.299	.092	.035	.131	5.05	3.21
*SD*	15.22	15.09	15.50	15.14	.157	.166	1274	1.02	.23	.48	.37	.16	.16	.25	.18	.12	.29	.46	.28	.45	.27	.18	.34	3.76	3.18
Girls																									
*N*	5,334	5,784	5,543	6,193	6,235	6,235	6,235	6,233	6,209	6,235	6,235	6,235	6,235	6,235	6,235	6,235	6,210	6,210	6,210	6,210	6,210	6,210	6,210	6,018	6,030
Mean	102.7	101.9	101.2	99.43	.483	.804	3402	.634	.262	10.67	.831	.029	.023	.073	.031	.013	.090	.288	.089	.275	.082	.032	.144	3.88	3.20
*SD*	14.32	14.24	14.04	14.42	.168	.174	1415	1.04	.266	.48	.37	.17	.15	.26	.17	.11	.29	.45	.28	.45	.27	.18	.35	3.24	3.07

Notes

Correlations for boys/girls are presented above/below the diagonal.

a *p *<* *.01.

Table 2 shows the results of the model predicting scores on each of the five decision‐making variables by the intercept and slope of intelligence after adjustment for confounders. (We excluded overall proportion bet from all analyses as it was very highly correlated [*r* = .962] with risk‐taking). As can be seen, intelligence was positively associated with risk adjustment and quality of decision‐making at age 11 in both boys and girls. In addition, in girls risk adjustment was positively related to IQ gains.

**Table 2:** Model results (standardized and unstandardized regression coefficients and standard errors) showing prediction of decision‐making by IQ (intercept and slope)

	Boys		Girls	
	β (SE)	*B* (*SE*)	β (*SE*)	*B* (SE)
Risk adjustment				
IQ intercept	.285 (.05)***	.026 (.01)***	.252 (.03)***	.025 (.00)***
IQ slope	.157 (.08)	.282 (.19)	.212 (.05)**	.282 (.09)**
Delay aversion				
IQ intercept	−.091 (.04)	−.002 (.00)	−.030 (.03)	−.001 (.00)
IQ slope	−.035 (.07)	−.013 (.03)	−.040 (.05)	−.013 (.02)
Risk‐taking				
IQ intercept	−.053 (.04)	−.001 (.00)	−.056 (.02)	−.001 (.00)
IQ slope	.039 (.08)	.011 (.02)	−.017 (.05)	−.004 (.01)
Quality of decision‐making				
IQ intercept	.228 (.03)***	.003 (.00)***	.242 (.03)***	.004 (.00)***
IQ slope	.037 (.06)	.011 (.02)	.120 (.05)	.026 (.01)
Deliberation time (seconds)				
IQ intercept	−.003 (.09)	.000 (.01)	−.049 (.03)	−.009 (.00)
IQ slope	.301 (.14)	.671 (.49)	.148 (.06)	.256 (.12)
*N*	6,279		6,235	

All estimates in the full model controlling for exact age, ethnicity, externalizing and internalizing problems, and maternal education.

**p < .01; ***p < .001.

## Discussion

Our study investigated the association between childhood intelligence and adolescent decision‐making measured with the Cambridge Gambling Task (CGT). In particular, it explored if decision‐making at the cusp of adolescence (age 11 years) was associated with the level and development of intelligence across childhood (ages 3–11 years). It showed that intelligence was positively related to the quality of decision‐making and risk adjustment, suggesting that there are significant, substantively, associations between intelligence and adapting behaviour to statistical risk. The association with quality of decision‐making suggests that those with a higher IQ were more likely than their counterparts to choose the likely outcome on more trials. Thus, they showed a tendency to make optimal choices more often than their counterparts. Risk adjustment, on the other hand, reflects reward‐seeking at high but not low probability ratios. Adjusting reward‐seeking behaviour in line with external contingencies is the rational strategy of showing a greater propensity to seek reward when the probability of obtaining it is high compared to when it is low. Therefore, rather than being prone or, conversely, averse to taking risks, adolescents with a higher IQ were rational and flexible decision‐makers, selecting higher bets when the chances of winning were favourable but lower bets when they were unfavourable. They were able to adjust their response to risky options in the light of information about outcome probabilities. Importantly, our study also showed that risk adjustment was associated with IQ *gains*, although only in girls. Several studies have shown that women show less risk adjustment (that is, they tend to have a less responsive betting style) on the CGT compared to men. Our findings could be taken to suggest that cognitive gains in girls translate into a more responsive betting style, but, in the absence of experimental data, theory‐driven hypotheses about gender‐specific effects and bigger effect sizes for the slope, we should be cautious about making such a statement. It would be very interesting, however, to explore in future research why cognitive gains and reward hyposensitivity are negatively associated in girls but are unrelated in boys.

By contrast, our study could not establish associations between intelligence and ‘risk‐proneness’ (i.e. risk‐taking) or delay aversion after adjustment for confounding. The absence of an association between intelligence and delay aversion may seem surprising. Delay aversion has been conceptualized as a motivational driver of impulsivity, associated negatively with intelligence. However, previous studies have suggested that delay aversion, as operationalized in the gambling task used here, does not distinguish the drive for immediate reward from the need to escape delay (Sørensen *et al*., 2017). This is seen as a crucial distinction as only the latter is related to a general tendency to avoid reflecting on problems, in turn associated negatively with intelligence. The lack of an association between intelligence and risk‐taking is not surprising. Risk‐taking on the gambling task used here is typically taken to approximate thrill‐seeking, inconsistently linked to intelligence (Steinberg, 2010).

Our findings, however, should be viewed in the light of an important limitation. In our study, ‘g’ was only theoretically inferred, given the cognitive assessments available in MCS. What may also concern some is our approach to capturing within‐individual change. That is, it could be suggested that our approach can only capture individual differences in intelligence and cannot possibly capture developmental ones (i.e. age‐related changes in intelligence) as we used age‐adjusted scores to calculate IQ at each sweep. We remind that we used standardized ability scores that had been adjusted for both item difficulty and age within sweep. This norming enables one, of course, to compare the performance of younger and older cohort children on a more level playing field within sweep. However, because MCS is a population‐based sample, a change in the relative position in the distribution of IQ over time (captured by the slope) does reflect true within‐individual change (developmental differences). (Of course, in any longitudinal sample the slope would reflect within‐individual change, but the relative position in the distribution would be sample‐specific. Because MCS is representative of the population, the estimates are true representations of the relative position in the distribution.)

In conclusion, our study suggests that children who show ineffective and inflexible decision‐making in adolescence (i.e. those showing poor skills in making optimal choices and adjusting responses to optimize outcomes in the face of changing probabilities) struggle cognitively compared to their peers. But is a change in IQ in childhood also important? It appears that this may be the case for risk adjustment in girls, for whom IQ gains were positively related to risk adjustment. Together, our findings suggest that intelligence cannot determine whether an 11 year old will be, in general, more (or less) risk‐prone or delay averse, at least on the computerized gambling task we used here. Rather, IQ (and IQ gains over time in girls) can predict effective and flexible decision‐making in early adolescence, when one's approach to making decisions can determine long‐term outcomes across many life domains.

## Footnotes

1 At ages 3, 5, and 11, the verbal assessments are on a 20‐80 metric, and at age 7, the verbal assessment is on a 55‐145 metric. The ‘linear stretch’ transformed the age 7 assessment to be on a 20‐80 metric, too. In each factor analysis (at ages 3, 5, and 7), the verbal assessment (on a 20‐80 metric) was identified as the first (anchoring) item. (‘Anchoring’ was not applicable to how IQ at age 11 was calculated, as there was only one cognitive assessment available at age 11. So the age 11 IQ was the assessment score standardised to have a mean of 100 and a standard deviation of 15.) This approach sets the latent metric for all ages to be identical (as the metric of the anchoring item is the same for all ages after the linear stretch), without forcing the means and variances to be the same (as standardizing does). We used the ‘verbal ability’ measure at each age to anchor as this was the only type of ability measured consistently in MCS across the four ages.
